# Antidepressant treatment, not depression, leads to reductions in behavioral and neural responses to pain empathy

**DOI:** 10.1038/s41398-019-0496-4

**Published:** 2019-06-07

**Authors:** Markus Rütgen, Carolina Pletti, Martin Tik, Christoph Kraus, Daniela Melitta Pfabigan, Ronald Sladky, Manfred Klöbl, Michael Woletz, Thomas Vanicek, Christian Windischberger, Rupert Lanzenberger, Claus Lamm

**Affiliations:** 1 Social, Cognitive and Affective Neuroscience Unit, Department of Basic Psychological Research and Research Methods, Faculty of Psychology, University of Vienna, Vienna, Austria; 2 Developmental Psychology Unit, Department of Psychology and Pedagogy, Ludwig Maximilian University, Munich, Germany; 3 Center for Medical Physics and Biomedical Engineering, Medical University of Vienna, Vienna, Austria; 4 Neuroimaging Labs, Department of Psychiatry and Psychotherapy, Medical University of Vienna, Vienna, Austria

**Keywords:** Molecular neuroscience, Depression, Human behaviour

## Abstract

Major depressive disorder (MDD) has been hypothesized to lead to impairments in empathy. Previous cross-sectional studies did not disentangle effects of MDD itself and antidepressant treatment. In this first longitudinal neuroimaging study on empathy in depression, 29 patients with MDD participated in two functional magnetic resonance imaging (fMRI) sessions before and after 3 months of antidepressant therapy. We compared their responses to an empathy for pain task to a group of healthy controls (*N* = 35). All participants provided self-report ratings targeting cognitive (perspective taking) and affective (unpleasant affect) aspects of empathy. To control for general effects on processing of negative affective states, participants additionally underwent an electrical pain task. Before treatment, we found no differences in empathic responses between controls and patients with MDD. After treatment, patients showed significant decreases in both affective empathy and activity of three a priori selected brain regions associated with empathy for pain. Decreases in affective empathy were moreover correlated with symptom improvement. Moreover, functional connectivity during the empathy task between areas associated with affective (anterior insula) and cognitive (precuneus) empathy decreased between sessions in the MDD group. Neither cognitive empathy nor responses to painful electrical shocks were changed after treatment. These findings contradict previous cross-sectional reports of empathy deficits in acute MDD. Rather, they suggest that antidepressant treatment reduces the aversive responses triggered by exposure to the suffering of others. Importantly, this cannot be explained by a general blunting of negative affect, as treatment did not change self-experienced pain.

## Introduction

Depression, one of the most widespread causes of disability of our time^1^, substantially impairs social functioning in various ways^2^. While the impact of major depressive disorder (MDD) on mood and basic emotional processing has been investigated intensely (see meta-analysis^3^), few attempts have been made to explore its influence on empathy, which is a crucial skill for everyday social interactions. Broadly defined, empathy entails isomorphic sharing of another person’s affective state, which can be elicited by either direct observation or imagination of the target’s emotion^4^ (see review for other definitions^5^). Evidence on the associations between depression and different behavioral and self-report measures of empathy has been reviewed by Schreiter et al.^6^. While their review clearly suggested a negative impact of depression on empathy, it also revealed problems of empathy research within depressed samples. First, and most importantly, the included studies were conducted on very heterogeneous samples that contained large proportions of patients undergoing antidepressant treatment: of the ten studies that specified medication status, four exclusively involved medicated patients, while the remaining six recruited mixed samples with 72% medicated patients on average. Unfortunately, analyses of medication intake effects were not conducted in any of the reviewed studies. Second, negativity biases such as negative self-perceptions of depressed patients might have had confounding effects on explicit measures such as self-report ratings and questionnaires^7,8^. For example, when patients with acute MDD were interviewed about a 4-month period prior to their depressive episode, they tended to over-report social maladjustment during the acute episode compared to after remission of symptoms^9^.

One way to overcome biases in self-report is the use of implicit measures of empathic responding, for example by using functional magnetic resonance imaging (fMRI) in combination with self-report and longitudinal observations. There is a substantial body of literature regarding the neural correlates of empathy for pain, consistently reporting mainly the anterior midcingulate cortex (aMCC) and the bilateral anterior insular (AI) cortex as being strongly engaged during empathic pain (see review^10^ and meta-analysis^11^). Notably, these areas are also part of a core network that is activated by the first-hand experience of pain. As of yet, the only (yet cross-sectional) fMRI study on empathy for pain in patients with MDD was carried out by Fujino et al.^12^. Consistent with previous behavioral studies, they found lower empathy ratings in response to empathy-inducing video clips in patients compared to controls. Furthermore, they reported reduced activity in the aMCC and right somatosensory cortex, which led them to the conclusion that patients with MDD exhibit a deficit in empathizing with others. However, this study included only medicated patients, restraining the authors from specifically relating these effects to MDD or to medication effects.

Such heterogeneity of samples becomes even more problematic in light of reported effects of acute administration of serotonergic antidepressants on various kinds of emotional processing (see meta-analysis and review^13^). These effects frequently emerge in research on functional connectivity. For example, escitalopram, a selective serotonin reuptake inhibitor and state-of-the-art first-line treatment for depression, was found to change effective connectivity during emotional face processing^14^, and even single doses influence functional connectivity and emotion regulation^15^. On top, the 5-HTTLPR polymorphism in the serotonin transporter gene has been found to have an influence on affective empathy^16^, underlining the important role of the serotonergic system in empathy, but also in social cognition in general^17^.

In this first longitudinal neuroimaging study on empathy in depression, we aimed at disentangling effects of untreated depression and antidepressant treatment on empathy. To this end, patients with MDD (*N* = 29) were scanned both before and after 3 months of serotonergic antidepressant treatment in two 7T fMRI sessions and were compared to a group of untreated healthy controls (HC) (*N* = 35). In both fMRI sessions, all participants completed an empathy task^18^, in which they watched short video clips of persons experiencing pain and provided self-report ratings of cognitive and affective empathy. In a second task, participants underwent painful electrical stimulation. This study design allowed us to distinguish between (a) effects due to the depressive disorder (at session 1) versus effects due to the psychopharmacological treatment (at session 2) and (b) effects that are specific to empathy versus domain-general in terms of processing of negative affective states (pain). If the impairments in empathy were due to untreated depression, then they should be evident at session 1 (before treatment). Moreover, if antidepressant treatment mitigates impairments of untreated depression, they should reduce from session 1 to session 2. However, if the previously reported impairments in empathy are actually due to the treatment, then they should emerge at session 2, but not at session 1. In addition, we tested for changes in connectivity between brain areas related to cognitive and affective empathy, as recent evidence showed enhanced connectivity during highly emotional situations^19^. Lastly, the employed empathy task allowed exploring possible effects of MDD and antidepressants on cognitive appraisal during empathizing^18^ (though no effect of appraisal was found in a recent study comparing inmates to HC^20^).

## Materials and methods

### Participants

Patients with acute MDD (18–50 years of age) were recruited from the outpatient clinic of the Department of Psychiatry and Psychotherapy, Medical University of Vienna. HC were recruited from the community. Participants gave written informed consent before participating. See Table 1 for sample characteristics. See Supplement M1 for details on recruitment and exclusion criteria. The study was approved by the Ethics Committee of the Medical University of Vienna and registered at ClinicalTrials.gov (NCT01477203). It was conducted in compliance with the Declaration of Helsinki^21^.

**Table 1:** Sample characteristics

Group	MDD	HC	*p*
*N*	29	35	
Age, years, mean ± S.E.M.	29.62 ± 1.76	27.41 ± 1.30	0.31^a^
Sex	21 females, 8 males	23 females, 12 males	0.58^b^
Age of onset (y)	21.4 ± 1.7	–	
Number of episodes (n)	3.4 ± 0.4	–	
Duration of disease (y)	9.6 ± 1.7	–	
Current episode/last episode remission (m)	6.5 ± 2.0	–	
Previous medication (unclear/yes/no)	3/14/12	–	
Handedness (r/l)	27/2	34/1	
Medication during study			
• Escitalopram (mg)	13.6 ± 1.1		
• Venlafaxine (mg)	109.6 ± 10.8		
• Mirtazapine (mg)	30^c^		
Citalopram plasma (ng/ml)	30.2 ± 7.6		
Venlafaxine plasma (ng/ml)	89.9 ± 16.9		
Treatment response^d^			
• Responder and remitter	16		
• Responder and nonremitter	6		
• Non-responder and nonremitter	7		

*MDD* Major depressive disorder patients, *HC* healthy control participants

^a^*t*-test

^b^χ^2^

^c^Only one patient

^d^Responder: ≥50% HAMD reduction to baseline; Remitter: HAMD <9

### Antidepressant treatment

All patients started their therapy with 10 mg escitalopram (a selective serotonin reuptake inhibitor, commercial name: Cipralex^®^) and were treated according to a flexible dose antidepressant treatment protocol. Patients who were not responding to the medication, changed therapy after 6–8 weeks to 75–150 mg venlafaxine (a selective serotonin-norepinephrine reuptake inhibitor; 13 of 29 patients). In case of intolerance to venlafaxine, 30 mg Mirtazapin (a noradrenergic and specific serotonergic antidepressant; one patient) was chosen as an alternative. Participants did not receive any other pharmacological treatment. To ensure that patients responded to the first-line treatment, Hamilton Depression Scale (HAMD-24^22^) scores were collected every second week.

### Experimental tasks and trial structure

#### Empathy task

**Fig. 1:**
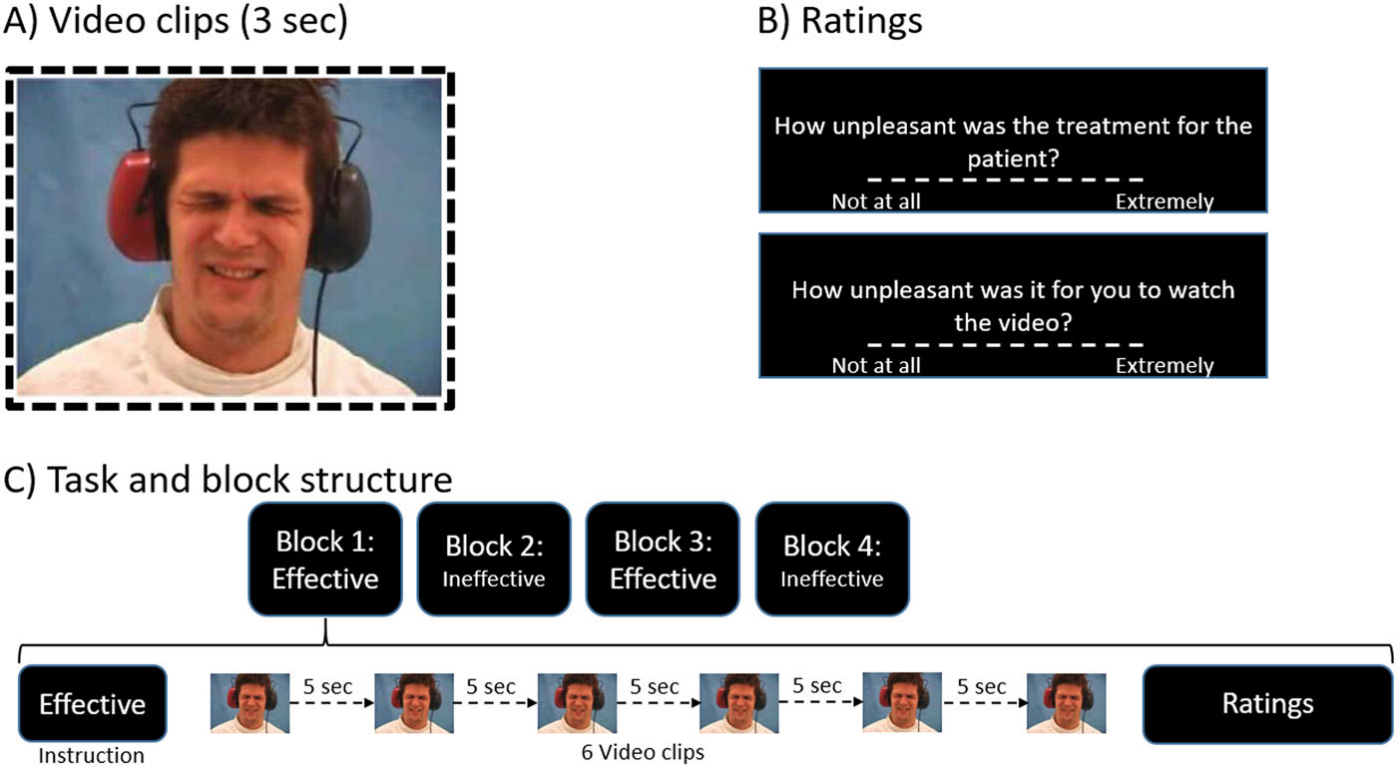
Empathy task description. In this task by Lamm et al.^18^, participants were told that they would view videos of tinnitus patients undergoing painful noise treatment. This treatment could be either effective or ineffective, depending on the instructions at the beginning of each block. Visual input was similar for effective and ineffective conditions (equally intense painful expressions; video clips counterbalanced across participants). **a** Example video clip: transition from neutral to painful expression within 3 s^18^. **b** Subjective ratings collected during the task: Target unpleasantness (upper part; cognitive empathy) and self-experienced unpleasantness (lower part; affective empathy rating). **c** Task and block structure: block order was counterbalanced across participants. After viewing initial instructions, participants saw six video clips in a row, separated by 5 s. The task had a mixed blocked-event related design, with each of the four blocks presenting only trials of the same category ("effective" or "ineffective"). At the beginning of each block, an instruction screen informed participants of whether the treatment for the patients that they were about to see had been effective or not. The block order, counterbalanced between participants, was either effective-ineffective-effective-ineffective or ineffective-effective-ineffective-effective. Each video was presented for 3 s and was followed by a 5 s intertrial interval, plus a 0–300 ms random jittering. Afterwards, ratings were collected via visual-analogue scales. Two 15-s baseline periods were recorded at the beginning and at the end of the task

An established empathy for pain task^18^ was employed (see Fig. 1). Participants viewed 24 video clips (duration: 3 s), each frontally showing the face of a single individual (12 f/12 m), whose facial expression transitioned from a neutral expression into a painful reaction in response to painful sound administration. Participants were told that the depicted people suffered from a neurological disease (tinnitus aurium), which was treated by repeatedly delivering intense auditory stimulation at specific frequencies through audiometric headphones. Allegedly, this therapy was very painful. Each video clip was shown once per session. The task lasted about 6:30 min and included four blocks of six stimuli each, divided into two experimental conditions: in the “effective” condition, participants were instructed that the therapy had been effective and thus the patients portrayed in the videos had recovered from their symptoms of disease; in the “ineffective” condition, they were told that the therapy had been ineffective. Twice per block, at random intervals, participants were asked to rate the degree of unpleasantness for the patients displayed in the videos (*target unpleasantness* rating), and the degree of unpleasantness for themselves when watching the videos and empathizing with the patients (*self-experienced unpleasantness* rating). These two types of ratings were obtained to measure both cognitive-evaluative (target unpleasantness) and affect-sharing (self-experienced unpleasantness) aspects of empathy^23,24^.

#### Electrical pain task

Participants also underwent an electrical pain task, as previously published^25,26^. This task consisted of short (500 ms) painful and nonpainful electrical stimuli delivered to the dorsum of the left hand. Due to technical problems, only 34 (of 35) HC and 26 (of 29) patients completed both sessions of this task. For a detailed task description, see Supplement M3.

### Experimental procedure

This study was a part of a larger project previously reported^25,26^. Participants completed the abovementioned tasks during two identical fMRI sessions, separated by 3 months. In between, participants in the MDD group underwent psychopharmacological antidepressant therapy (see Supplement M2 for details).

### Questionnaires

HAMD, Interpersonal Reactivity Index (IRI^27^), Emotion Contagion Scale (ECS-D^28^) and Emotion Regulation Questionnaire (ERQ, German version^29,30^) scores were obtained during both sessions. HAMD changes were examined with a two-tailed *t*-test for paired samples. Pearson correlations of HAMD improvement scores with changes in behavior and brain activity across sessions were computed. Questionnaire scales were separately analyzed with repeated measures ANOVAs with the between-subjects factor *group* (MDD versus HC) and the within-subject factor *session* (S1 versus S2). In case of significant main effects or interactions, post hoc independent samples (for between-groups comparisons) or paired samples (for between-sessions comparisons) *t*-tests were carried out.

### Behavioral data analysis

The two types of ratings in the empathy task (target unpleasantness, self-experienced unpleasantness; see Fig. 1) were analyzed separately with two mixed-model ANOVAs with the within-subjects factors *condition* (effective versus ineffective) and *session* (S1 versus S2) and the between-subjects factor *group* (MDD versus HC). Post hoc comparisons were carried out in case of significant main effects or interactions.

### fMRI data acquisition and analysis

Image acquisition and preprocessing are detailed in Supplement M4. First-level and second-level analyses were performed with SPM12 (Wellcome Trust Centre for Neuroimaging, http://www.fil.ion.ucl.ac.uk/spm), adopting a general linear model approach. In the empathy task, the first-level design matrix of each subject contained four regressors: “effective” videos, “ineffective” videos, ratings, and instructions. In the electrical pain task, first-level regressors comprised four stimulation (pain, no-pain, uncertain pain, uncertain no-pain) and three anticipation (certain pain, certain no-pain, uncertainty) conditions. Anticipation was not of interest and therefore orthogonalized to the respective stimulation regressors. Regressors were convolved with the canonical hemodynamic response function and its temporal and dispersion derivatives. White matter and cerebrospinal fluid signals were extracted before smoothing and used as nuisance signals together with six realignment parameters.

#### Empathy task

For our main analysis, parameter estimates were extracted (REX toolbox: http://web.mit.edu/swg/software.htm) from all participants and conditions in 10-mm-spherical ROIs, centered on three clusters reported in the meta-analysis of Lamm et al.^11^: aMCC (coordinates: *x* = −2 *y* = 23 *z* = 40), left anterior insula (lAI; −40 22 0), and right anterior insula (rAI; 39 23–4). Their sensitivity to experimental manipulations affecting empathy had been previously shown^31^. Effective > baseline and ineffective > baseline first-level contrasts of both sessions were used. These values were entered into a four-way mixed-model ANOVA, in which we tested whether the experimental factors (*session*: 1 versus 2; *condition*: effective versus ineffective; *ROI*: lAI versus aMCC versus rAI; *group*: MDD versus HC) showed any significant effects. In case of such differences, post hoc comparisons were carried out. Complementary whole-brain analysis approach and detailed results, as well as task validation are reported in the Supplement (Supplement R1; Supplemental Tables T1–3).

#### Empathy task: functional connectivity analysis

This analysis aimed at assessing treatment-induced changes in task-dependent connectivity over time in patients with MDD. Using the generalized form of context-dependent psychophysiological interactions (gPPI^32^), this analysis focused on the three predefined regions of interest (lAI, rAI, aMCC) as seed regions. We were interested in connectivity changes over time (S2 < S1, S2 > S1) in both groups separately and in contrast to each other. On top of connectivity analyses on the pooled data across conditions, we explored connectivity changes within the single conditions (effective, ineffective). Physiological activity of each subject in the three ROIs was defined as the principal eigenvariate of a 10-mm-radius sphere centered on meta-analysis coordinates^11^. Single-subject PPI contrast images were then entered into one-sample/two-sample *t*-tests at the second level. To perform family-wise control for Type I errors, Bonferroni correction for the number of tests was applied. Results were therefore considered significant at *p* < 0.00185.

#### Electrical pain task

Parameter estimates were extracted from the same 10-mm spherical ROIs (lAI, rAI, aMCC). Pain > baseline and no-pain > baseline first-level contrasts of both sessions and groups were entered into a four-way mixed-model ANOVA including the factors *session* (1 versus 2), *intensity* (pain versus no-pain), *ROI* (lAI versus aMCC versus rAI) and *group* (MDD versus HC). In case of significant main effects or interactions, post hoc comparisons were carried out. For the electric pain intensity values, a mixed-model ANOVA with factors *session* (S1 versus S2), *intensity* (pain versus no-pain) and *group* (MDD versus HC) was run (see Supplement R2 and Supplemental Table T4). For the complementary whole-brain analysis of this task, see Kraus et al.^33^.

### Medication dosage, change of treatment, and additional control analyses

Pearson correlations between ratings/extracted values and medication dosage (dose equivalents computed according to Hayasaka et al.^34^) were computed. Differences between differently treated groups (12 weeks escitalopram versus 6/8 weeks escitalopram followed by venlafaxine/mirtazapine) regarding changes in self-report ratings/extracted values over time were tested with independent samples *t*-tests. The following additional control analyses were run (Supplement R4–6): Pearson correlations between ratings/extracted values and post-treatment HAMD scores (testing whether remission accounted for differences in empathy) and number of episodes/duration of disease (testing for an influence of MDD history on empathy) were computed. Improvement in ERQ Reappraisal was included as covariate in the main imaging and behavioral analysis.

## Results

### Treatment response

Patients showed significant improvement in HAMD scores over time (*t*(28) = 13.32, *p* < 0.001; mean scores ± S.E.M.: S1: 25.93 ± 1.19; S2: 8.38 ± 1.13). See Table 1 for detailed clinical outcomes.

### Questionnaires on empathy and emotion

Mean values of pre- and post-treatment questionnaire scores ± S.E.M., as well as *p*-values of post hoc tests are listed in Table 2. Patients showed between-session improvement in ERQ reappraisal and consistently higher values in IRI personal distress. Emotional contagion by sadness decreased significantly between sessions in patients. ANOVA results are reported in Supplement R5.

**Table 2:** Questionnaire data of both groups and both sessions. Values are mean ± S.E.M. Post hoc tests were carried out in case of significant main effects or interactions and represent Benjamini–Hochberg-adjusted *p*-values of paired (within-group between sessions) or independent (between-group within sessions) *t*-tests (bold if significant). Additional *t*-test *p*-values of the remaining questionnaire scales (no ANOVA effects) are reported as well

Questionnaire scale	Healthy controls		Patients with MDD		Post hoc test results			
	S1	S2	S1	S2	MDD S1 versus S2	MDD versus HC S1	MDD versus HC S2	HC S1 versus S2
ERQ reappraisal	28.97 ± 1.24	28.97 ± 0.89	24.18 ± 1.52	27.04 ± 1.09	***p*** **=** **0.046**	***p*** **=** **0.046**	*p* = 0.167	*p* = 1.00
ERQ suppression	11.45 ± 0.92	11.45 ± 0.83	15.63 ± 1.13	15.18 ± 1.01	*p* = 0.739	***p*** **=** **0.008**	***p*** **=** **0.018**	*p* = 1.00
ECS fear	8.67 ± 0.33	8.35 ± 0.32	10.23 ± 0.38	9.11 ± 0.37	***p*** **=** **0.036**	***p*** **=** **0.016**	*p* = 0.075	*p* = 0.385
ECS joy	13.02 ± 0.28	13.11 ± 0.27	11.00 ± 0.32	11.50 ± 0.31	*p* = 0.142	***p*** **<** **0.001**	***p*** **=** **0.001**	*p* = 0.724
ECS love	12.79 ± 0.39	12.79 ± 0.34	11.00 ± 0.45	11.57 ± 0.39	*p* = 0.129	***p*** **=** **0.022**	*p* = 0.084	*p* = 1.00
ECS anger	8.38 ± 0.35	8.79 ± 0.34	9.61 ± 0.40	9.57 ± 0.39	*p* = 0.918	*p* = 0.062	*p* = 0.115	*p* = 0.182
ECS sadness	8.58 ± 0.35	8.97 ± 0.37	10.03 ± 0.40	9.11 ± 0.42	***p*** **=** **0.012**	***p*** **=** **0.016**	*p* = 0.562	*p* = 0.083
IRI empathic concern	14.67 ± 0.37	14.38 ± 0.41	14.73 ± 0.42	15.03 ± 0.47	*p* = 0.530	*p* = 0.986	*p* = 0.147	*p* = 0.449
IRI fantasy	13.00 ± 0.51	13.35 ± 0.49	13.23 ± 0.59	12.92 ± 0.56	*p* = 0.425	*p* = 0.637	*p* = 0.658	*p* = 0.292
IRI personal distress	8.64 ± 0.47	8.79 ± 0.39	12.92 ± 0.53	12.26 ± 0.45	*p* = 0.191	***p*** **<** **0.001**	***p*** **<** **0.001**	*p* = 0.719
IRI perspective taking	15.73 ± 0.51	15.64 ± 0.42	15.30 ± 0.58	14.73 ± 0.48	*p* = 0.130	*p* = 0.565	*p* = 0.201	*p* = 0.807

### Behavioral data

The ANOVA on *self-experienced unpleasantness* ratings yielded a significant *condition* main effect, *F*(1,62) = 16.12, *p* < 0.001, *η*^*2*^_*p*_ = 0.206: participants reported more self-experienced unpleasantness in the ineffective as compared to the effective condition (Mean ± S.E.M. = 50.07 ± 2.28 and 46.83 ± 2.26, respectively). Also, a main effect of *session* (*F*(1,62) = 4.24, *p* = 0.044, *η*^*2*^_*p*_ = 0.064) was found, as well as an interaction of *session* × *group* (*F*(1,62) = 7.62, *p* = 0.008, *η*^*2*^_*p*_ = 0.110). Post hoc *t*-tests (mean values of effective and ineffective condition) showed that this effect was driven by a significant decrease in unpleasantness ratings in the MDD group (S1: 51.64; S2: 42.07; *t*(28) = 3.651, *p* = 0.001, *Cohen’s d* = 1.37). No such decrease was observed in the HC group (S1: 49.35; S2: 50.75; *t*(34) = −0.53, *p* = 0.597). Post hoc *t*-tests showed no difference between MDD and HC before treatment (*t*(62) = −0.46, *p* = 0.644, *Cohen’s d* = 0.11), but a borderline significant difference after treatment (*t*(62) = 1.99, *p* = 0.050, *Cohen’s d* = 0.51). The *condition* × *session* × *group* interaction remained nonsignificant (*p* = 0.216). None of the other main effects and interactions was significant (all *p*-values > 0.192).

**Fig. 2:**
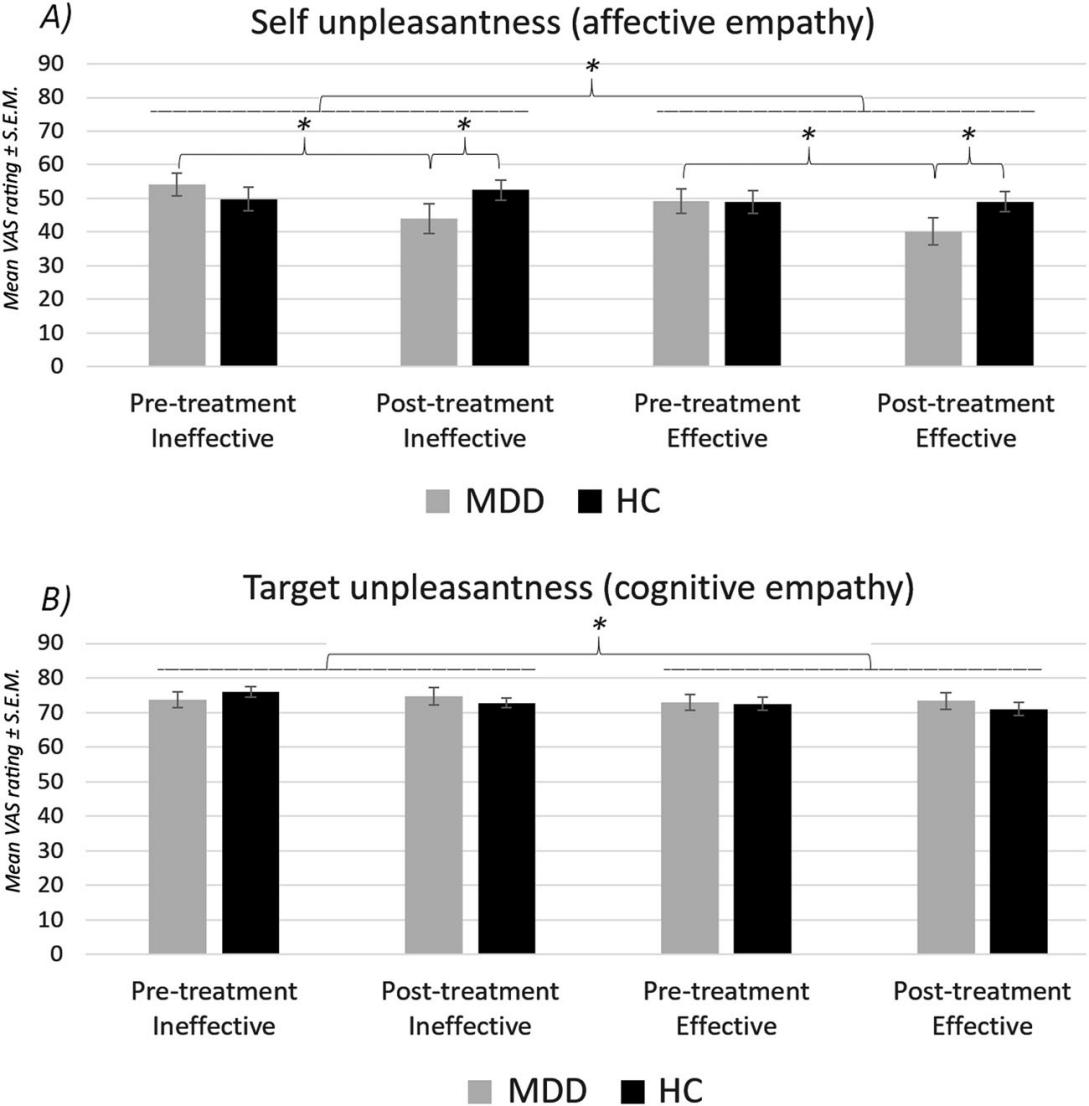
Empathy task—behavioral results. Rating results show (**a**) a significant decrease in self-experienced unpleasantness ratings in the MDD group in both the ineffective condition and the effective condition, and (**b**) no significant differences or changes in target unpleasantness ratings. Values are mean ratings ± S.E.M. Pre-treatment = session 1, Post-treatment = session 2. Asterisks mark significant differences

The ANOVA on *target unpleasantness* ratings yielded a significant main effect of *condition* (*F*(1,62) = 6.11, *p* = 0.016, *η*^*2*^_*p*_ = 0.090), which was due to higher target unpleasantness ratings in the ineffective as compared to the effective condition (Mean ± S.E.M. = 74.26 ± 1.21 and 72.48 ± 1.28, respectively). The remaining main effects and interactions were not significant (all *p*-values > 0.209). See Fig. 2 for illustration of behavioral results.

**Fig. 3:**
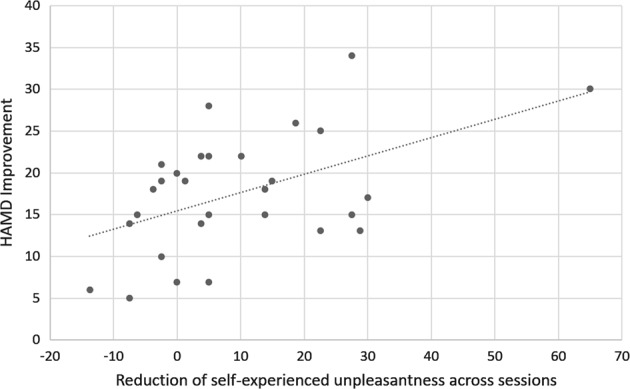
Correlation analysis. Correlation of symptom improvement and reductions in self-experienced unpleasantness (affective empathy) across sessions. Decreases in HAMD scores (S1-S2) correlated significantly with reductions in self-experienced unpleasantness (*r* = 0.50, *p* = 0.006)

HAMD improvement (S1-S2) correlated significantly with reductions in self-experienced unpleasantness (S1-S2, “affective empathy”): *r* = 0.50, *p* = 0.006 (stronger improvement in symptom severity was related to higher reduction in unpleasantness; see Fig. 3). Including treatment change (*r*_*cov*_ = 0.58, *p* = 0.001) or dosage (*r*_*cov*_ = 0.50, *p* = 0.006) as covariates in this analysis did not substantially change these results. Differences in target unpleasantness (*r* = 0.347, *p* = 0.065) and brain activity (all *p*-values > 0.89) across sessions were not significantly correlated to HAMD improvement.

No significant correlations between medication dosage and self-report ratings were found (all *p*-values > 0.281). Change of medication after 6/8 weeks did not have a significant effect on self-report ratings (all *p*-values > 0.529).

### fMRI data

#### Empathy task

**Fig. 4:**
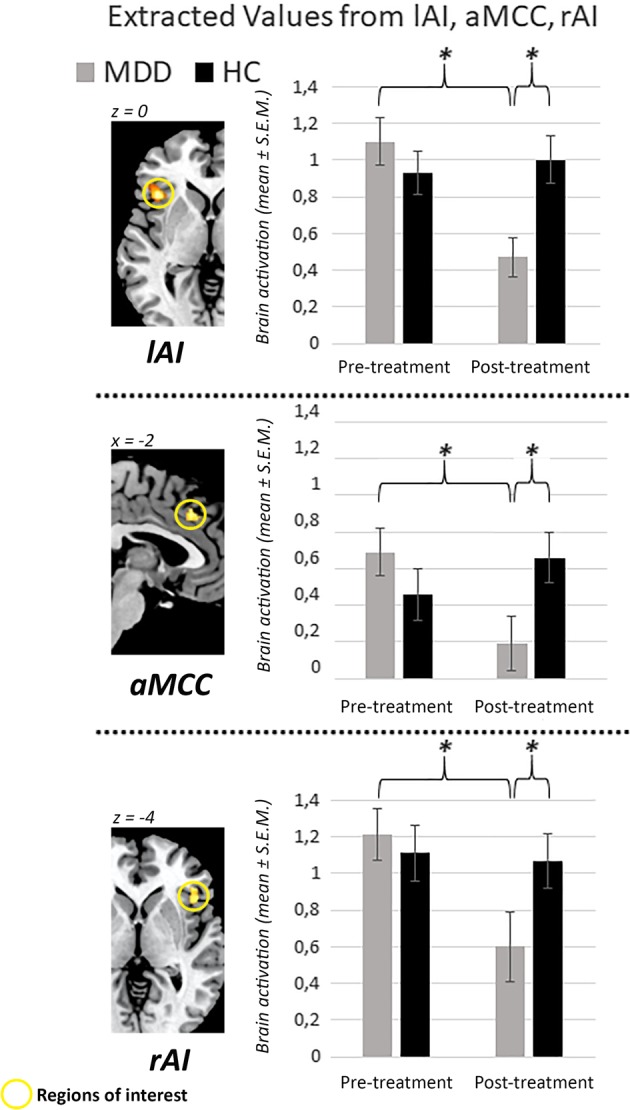
Empathy task—fMRI results. Extracted values (mean across effective and ineffective condition; highly similar values were found in both conditions) from all three ROIs show a similar pattern of results: decreased values in response to empathic pain across sessions in the MDD group, as well as a significant difference between HC and MDD in session 2 only. Left side of the figure: activation maps for illustration purposes, displaying the spatial distribution of brain activity within the ROIs (session 2: HC > MDD). Values are mean extracted beta values ± S.E.M. lAI = left anterior insula, rAI = right anterior insula, aMCC = anterior midcingulate cortex. S1 = session 1, S2 = session 2. Asterisks mark significant differences

The four-way mixed-model ANOVA revealed significant main effects of *session* (*F*(1,62) = 5.75, *p* = 0.019, *η*^*2*^_*p*_ = 0.085) and *ROI* (*F*(2,124) = 21.65, *p* < 0.001, *η*^*2*^_*p*_ = 0.259). The main effect of *ROI* was driven by significantly lower activity in the aMCC (0.533 ± 0.077) as compared to both insular ROIs (lAI: 0.896 ± 0.066, rAI: 1.012 ± 0.092). Importantly, a significant interaction effect of *session* × *group* (*F*(1,62) = 7.70, *p* = 0.007, *η*^*2*^_*p*_ = 0.110) was found. This interaction was driven by a strong decrease in activation in the MDD group from the first to the second session (mean ± S.E.M.: S1: 0.996 ± 0.119, S2: 0.439 ± 0.132). This was not the case in the HC group (mean ± S.E.M.: S1: 0.890 ± 0.108, S2: 0.930 ± 0.121). Post hoc *t*-tests (mean values of effective and ineffective condition across ROIs) showed no difference between MDD and HC before treatment (*t*(62) = −0.66, *p* = 0.509), but a significant difference after treatment (*t*(62) = 2.74, *p* = 0.008, *Cohen’s d* = 0.69), as well as a significant between-sessions difference for the MDD group (*t*(28) = 3.26, *p* = 0.003, *Cohen’s d* = 1.23). None of the other main effects or interactions were significant (all *p*-values > 0.149). See Fig. 4 for illustration of fMRI ROI results. See Supplement R1 for the complimentary whole-brain analysis of this task. In short, this analysis revealed significant between-session decreases in lAI, aMCC, supplemental motor area and occipital cortex in the MDD group (MDD S1 > S2). We found higher activity in the secondary somatosensory cortex (SII) in the MDD group (compared to HC) in both sessions. No differences were observed in the interaction contrasts (e.g., HC > MDD: S1 > S2).

No significant correlations between medication dosage and brain activity were found (all *p*-values > 0.261). Change of medication after 6/8 weeks did not have a significant effect on extracted values (all *p*-values > 0.782).

### Functional connectivity in the empathy task

MDD treatment effect (MDD S1 > S2): using the lAI as seed region, decreased connectivity to the precuneus (peak: *x* = −12 *y* = −50 *z* = 40, *k* = 1224, cluster-level *p*_*FWE-corr*_ < 0.001) was found in the ineffective condition in the post-treatment session contrasted to the pre-treatment session. In the effective condition, as well as in all contrasts using the other ROIs as seed regions, no connectivity changes across sessions were found. In the pooled analysis, decreased connectivity to the right occipital cortex (peak: *x* = 6 *y* = −96 *z* = 9, *k* = 5759, cluster-level *p*_*FWE-corr*_ < 0.001) and right inferior parietal lobule (peak: *x* = 48 *y* = −70 *z* = 40, *k* = 764, cluster-level *p*_*FWE-corr*_ = 0.001) were found. The HC group showed increased connectivity between lAI and occipital cortex in the second compared to the first session. Clusters are reported in Table 3.

**Table 3:** Significant clusters of gPPIs in both effective and ineffective condition, using the three regions of interest as seed regions

Contrast	Seed	Cluster	*k*	Peak *x*	Peak *y*	Peak *z*	*p*_*FWE-corr*_	Region
*Ineffective condition*								
MDD S1 > S2	lAI	1	1224	−12	−50	40	<0.001	Precuneus
	rAI	–						
	aMCC	–						
MDD S2 > S1	No surviving clusters for any of the seed regions							
HC S1 > S2	No surviving clusters for any of the seed regions							
HC S2 > S1	lAI	1	2345	10	−79	6	<0.001	R occipital cortex
	rAI	–						
	aMCC	–						
MDD > HC (S1 > S2)	lAI	1	9038	−33	−54	−6	<0.001	L fusiform gyrus
	rAI	1	2099	8	−81	1	<0.001	R occipital cortex
*Effective condition*								
No surviving clusters for any of the contrasts								
*Pooled (effective* *+* *ineffective)*								
MDD S1 > S2	lAI	1	5759	6	−96	9	<0.001	R occipital
		2	764	48	−70	40	0.001	R inferior parietal lobule
	rAI	–						
	aMCC	–						
MDD S2 > S1	No surviving clusters for any of the seed regions							
HC S1 > S2	No surviving clusters for any of the seed regions							
HC S2 > S1	lAI	1	2345	10	−79	6	<0.001	R occipital
	rAI	–						
	aMCC	–						
MDD > HC (S1 > S2)	lAI	1	16197	−10	−84	−5	<0.001	L lingual gyrus
		2	1013	−40	−58	−9	<0.001	L fusiform gyrus
	rAI	1	4914	−27	−81	16	<0.001	L occipital
	aMCC	1	968	−12	−81	−5	<0.001	L occipital
HC > MDD (S1 > S2)	No surviving clusters for any of the seed regions							

Group comparison (MDD S1 > S2) > (HC S1 > S2): using the lAI as seed region, decreases in connectivity with the fusiform gyrus and the lingual gyrus (see Table 3) were found. For the other seed regions, changes in connectivity to the occipital cortex were found.

### Electrical pain task

**Fig. 5:**
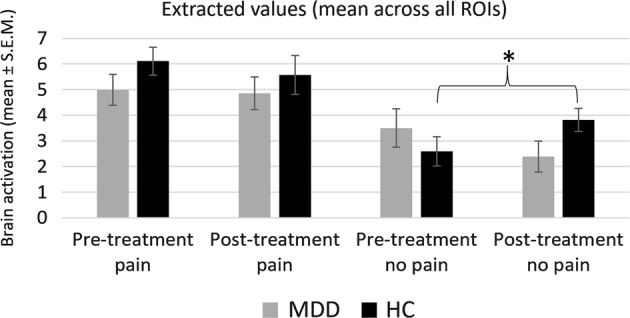
Electrical pain task—fMRI results. Extracted values (mean across all three ROIs ± S.E.M.) show no differences in processing of painful stimuli, but an increase in values in response to nonpainful stimuli across sessions only in the healthy controls. See Supplement R2 for separate ANOVAs of single ROIs, yielding similar results

The four-way mixed-model ANOVA revealed significant main effects of *intensity* (*F*(1,58) = 25.23, *p* < 0.001, *η*^*2*^_*p*_ = 0.303; higher values in response to pain compared to no-pain) and *ROI* (*F*(1,58) = 38.94, *p* < 0.001, *η*^*2*^_*p*_ = 0.402). We also found a significant interaction of *ROI* × *intensity* (*F*(1,58) = 18.01, *p* < 0.001, *η*^*2*^_*p*_ = 0.237), driven by a smaller difference between pain and no-pain in the aMCC, as compared to the other ROIs (left AI, right AI; see Supplement). In addition, a *session* × *intensity* × *group* (*F*(1,58) = 4.95, *p* = 0.030, *η*^*2*^_*p*_ = 0.079) three-way interaction was found, mainly driven by the HC group showing a between-sessions increase in values in response to no pain stimuli, which was also confirmed in a post hoc paired *t*-test (mean ± S.E.M.: S1: 2.604 ± 0.572, S2: 3.826 ± 0.457; *t*(33) = −2.06, *p* = 0.047). All other post hoc *t*-tests regarding this interaction remained nonsignificant (all *p*-values > 0.062). See Fig. 5 for illustration of fMRI ROI results. See Supplemental table T4 for calibration values.

## Discussion

In this study, we aimed at overcoming limitations of previous behavioral and neuroimaging work on empathy in MDD. By measuring an untreated sample at baseline and follow-up after 3 months of antidepressant therapy, we aimed to disentangle the effects of MDD and antidepressant treatment on empathy. By controlling for neural responses in an electrical pain task, we are also able to draw conclusions regarding the specificity of the observed effects for empathy versus affective responding to aversive stimuli in general.

Contrary to previously reported evidence, neither behavioral nor neural differences between patients with MDD and HC were observed in the first fMRI session, indicating a “normal” empathic response in patients with acute MDD before they underwent antidepressant treatment. After 3 months of therapy, patients showed decreased neural responses in a priori selected brain areas that are reliably activated by empathic pain (bilateral AI and aMCC), and reported reduced self-experienced unpleasant affect in response to the pain of others. This reduction is significant both when compared to their own pre-treatment responses and when compared to those of the HC in the post-treatment session. Here, it needs to be noted that the complimentary whole-brain analysis revealed similar decreases within the MDD group. Anyhow, no differences appeared in the interaction contrasts (e.g., HC > MDD: S1 > S2). Decreases of self-experienced unpleasantness were correlated with symptom severity reduction (HAMD scores). At first sight, these results contradict previous findings in terms of both behavioral (see review^6^) and neural responses^12^. However, taking into consideration that previous findings were mainly derived from medicated samples, they are well in line with the present post-treatment results. Insufficient inclusion of nonmedicated participants and lack of follow-up analyses on medication effects in previous studies restrained their authors from drawing reliable conclusions on the “pure” effects of MDD on empathy. To our knowledge, the present study is the first to show the behavioral and neural effects of (a) untreated MDD and (b) antidepressant treatment on empathy.

Special attention should be dedicated to the comparison of our results with those reported by Fujino et al.^12^, the only previous systematic fMRI investigation of empathy in patients with MDD. Comparability is strongly enhanced because both studies used video clips as stimuli (faces of patients showing pain in the present study, hands being harmed in Fujino et al.). The present post-treatment results of lowered empathy ratings as well as reduced activation in the aMCC parallel similar findings in the medicated sample of Fujino et al.^12^. Opposed to the findings of this previous study, patients did not show reduced, but enhanced activity in the SII compared to HC in both sessions. However, there was no significant change in SII activity in the MDD group between sessions. SII has been reported to play an important role in encoding vicarious pain perception^35^. It is specifically recruited in people who experience localized sensations of vicarious pain^36,37^. Thereby, enhanced activity in comparison to HC may stem from a more vivid or self-referential representation of others’ pain in MDD. Future studies might explore a possible relationship of vicarious sensory pain experience and MDD.

Differentiation between the two distinct behavioral ratings used in this study is another important aspect of our results: the target unpleasantness rating, which is a rather cognitive-evaluative measure of others’ pain, was unaffected by both MDD and antidepressant treatment. On the contrary, the self-related unpleasantness ratings, which reflect vicarious distress (for detailed views on this differentiation, see^23,24^), were significantly reduced over time in the MDD group. On top, these reductions were highly correlated to symptom severity improvement. Thus, antidepressant treatment seems to have a protective function for the affective processing of negative events in a social context. Cognitively, patients were similarly aware of the extent of pain that the tinnitus patients expressed, but their own affective state was less affected. This differentiation of effects on cognitive versus affective empathy is in line with previously reviewed findings^38^, which were thought to be linked to MDD instead of antidepressant treatment. If the decrease in self-experienced unpleasantness was just a function of improved mood and resulting avoidance or reinterpretation of mood-incongruent events, an effect on the cognitive evaluation of others’ unpleasantness would be expected as well. We interpret these findings in terms of a recently proposed framework by Coll et al.^39^, who argued that specific manipulations could independently affect either the ability of an individual to accurately perceive another’s emotional state (*emotion identification*) or their degree of *affective sharing* of that state. We did not observe an effect of antidepressant treatment on emotion identification (target unpleasantness evaluation), but found an effect on empathy in terms of affectively sharing the other’s emotions (self-experienced unpleasantness). Usage of rating measures targeting different aspects of empathy should be considered in future clinical studies on empathy.

Self-reported improvements in ERQ and ECS, as demonstrated by partial normalization towards levels of HC in the post-treatment session, point to the known beneficial effects of antidepressant treatment on emotion regulation^40^. The fact that treatment led patients to (a) having a stronger tendency to reappraise situations for better emotion regulation and (b) being less likely to catch up others’ negative affect is in good accordance with less self-experienced unpleasantness in the empathy task. Similar to previous reports^38,41,42^, we observed significantly higher values of IRI personal distress (measuring feelings of discomfort that occur as a result of observing another’s negative experience^27^) in depressed patients, which was not changed by treatment. This discrepancy between trait and state distress might be explained by the relatively general nature of the items of the IRI (e.g., “I tend to lose control during emergencies”). Also, the personal distress trait appears to be rather stable even over long time periods^43^, while situation-specific distress (e.g., encountering someone in pain) is apparently more prone to interventions such as antidepressant treatment.

Contrary to behavioral findings (higher unpleasantness ratings in response to ineffective treatment condition) and previous evidence for the influence of appraisal on neural responses to empathy^18^, no effect of cognitive appraisal (effective versus ineffective condition) was found in the main imaging analysis. In the present study, only 12 trials per condition were used, while previously^18^, 20 trials per condition were shown. This relative lack of power represents a likely reason for absence of an effect. Nevertheless, analyses of psychophysiological interactions showed differentiation also on the neural level, with significantly reduced post-treatment connectivity between lAI and precuneus only in the ineffective condition. Admittedly, these results need to be interpreted with caution, because connectivity changes across sessions were only significant within the MDD group, not when contrasting them with HC. The AI has consistently been associated with affect sharing^10^, while the precuneus is involved in rather cognitive functions implicated in empathy, such as perspective taking or theory of mind^44,45^. Previous research demonstrated enhanced interplay between brain areas involved in affective versus cognitive empathy under conditions of high negative emotionality^19,46^. Restriction of the effect to the ineffective condition might therefore be explained by its higher negative emotionality (as reflected in subjective ratings). Interestingly, connectivity between insular ROIs and occipital regions decreased in the MDD group across sessions (compared to HC). As both visual occipital regions and insular cortices are implicated in facial emotion processing^47^, reduced interplay between these regions might be related to the decreased affective impact of others’ pain in the MDD group.

Interestingly, no relevant effects on pain processing (apart from an increase in no-pain brain activity in the HC group, which was probably driven by slightly, yet not significantly different calibration values between sessions in this group) were observed in the ROIs. This speaks against general blunting of affective responses, as well as against generally decreased neural reactions to negative stimuli, but rather for an effect that is specific to affective responding in a socioemotional context. Since we did not obtain self-report ratings in the pain task, however, changes in the subjective experience of pain cannot be evaluated. Slightly lowered sample size in the electrical pain task in comparison to the empathy task also represents an obvious limitation.

Our study was not a full randomized controlled trial, which restrains us from excluding second-order effects as an alternative explanation for the observed changes after treatment. Changes in empathic responding after treatment could be directly related to the influence of antidepressants on the serotonergic (but possibly also the noradrenergic^48^) system. An alternative explanation, which is in line with the cognitive neuropsychological theory of antidepressant drug action^49^, suggests reversal of depression-related negative affective bias by antidepressants. Usually, depressed patients show a bias towards negative stimuli in simple emotion processing tasks^50,51^. Antidepressants lead to normalization of such biases, demonstrated for example by reduced neural responses to negative facial expressions^52,53^. Such reduced responses to negative affective experiences might also come into play in more complex social situations involving empathy. Influences of SSRIs on the hemodynamic response (e.g., via changes in blood flow) could be seen as a potential limitation. However, in light of recent evidence that did not show effects of SSRIs on brain hemodynamic responses nor on vascular artifacts, this seems unlikely as an alternative explanation^54,55^.

While application of three different antidepressants may be considered disadvantageous in terms of homogeneity, this allowed maximization of treatment effectivity for the patients, and thus also reflects a more ethical and realistic scenario compared to a more controlled design. Importantly, change of medication did not have significant effects on behavioral or neural measures.

Our results should be taken into consideration by future studies investigating clinical samples. For example, recent studies in samples under treatment reported depression-related deficits in emotion recognition (see meta-analysis^56^) and reading of facial expressions^57^. This is not to say that such findings are solely related to antidepressant treatment (see contrary evidence^58^). Still, interactions may play a role in such cases, and care needs to be taken before ascribing specific deficits to groups of patients without sufficiently controlling for medication status.

The presented insights put a different complexion on depression-related changes of empathy. As demonstrated, antidepressant treatment might lead to effects that were previously attributed to MDD. Considering the observed relationship between reductions in affective empathy and improvements in symptom severity, this might be an advantageous side effect with protective function, which could possibly spread to other kinds of negative events in social contexts. It remains to be explored whether these treatment-induced changes also lead to changes in prosocial behavior (see reviews^59–61^ for discussion of the relationship between empathy and prosocial behavior; recent research showed unchanged moral judgements in medicated patients with MDD^62^, but increased cooperation in symptom-free patients with a history of MDD^63^): on the one hand, lowered empathic distress might enable medicated patients to assign more cognitive resources to helping another person in need (here, it has to be emphasized again that the cognitive evaluation of the others’ pain was left unchanged). On the other hand, it might substantially reduce the salience of the situation, and, consequently the motivation to help the other. Thus, this seems to be an important endeavor for future research.

## Supplementary information

Supplemental Information
