# Supplementary material for: Antidepressant treatment, not depression, leads to reductions in behavioral and neural responses to pain empathy
Source: Transl Psychiatry. 2019 Jun 7;9:164. doi: 10.1038/s41398-019-0496-4 (PMC6555809; doi:10.1038/s41398-019-0496-4)
Supplement: Supplementary file 1 — Supplemental Information [file 41398_2019_496_MOESM1_ESM.docx]

Antidepressant treatment, not depression, leads to reductions in behavioral and neural responses to pain empathy

**Supplemental Information**

**Section M - Supplemental Materials and Methods**

**M1 – Participant Recruitment and Exclusion Criteria**

Patients with MDD were diagnosed based on the Structured Clinical Interview for DSM-IV for Axis I and Axis II disorders (1, 2), to ascertain the presence of MDD and exclude psychiatric axis I and II comorbidities. All patients reported moderate to severe symptoms based on the Hamilton Depression Rating Scale (3) and were treatment free in the three months preceding the start of the experiment. Patients with internal or neurological disorders, as well as pregnant or breastfeeding women, were excluded from the study. Healthy control (HC) participants (inclusion criteria: aged 18-50 years; drug-free; no history of relevant somatic, neurological or any former or current psychiatric disorder) were recruited via local advertisements and postings at public places in the General Hospital of Vienna. To exclude drug abuse, urine tests were performed in all participants and sessions. Participants received a compensation of € 150 (+ max. € 50 depending on performance in an unrelated task) for taking part in the study. A-priori power calculations with G*Power (4) yielded a minimum sample size of 28 for detecting a medium effect of *f*=.025 (given a statistical power of 0.8, alpha = .05).

**M2 – Antidepressant Treatment**

***Treatment Response***

The HAMD scores on MRI sessions 1 & 2 were compared and symptom severity improvement was examined with a two-tailed *t*-test for paired samples. HAMD symptom level means are available upon request.

**M3 – Experimental Tasks**

Task stimulus presentation and response collection were performed using Cogent 2000 (developed by the Cogent 2000 team at the FIL and the ICN) and Cogent Graphics (developed by John Romaya at the LON at the Wellcome Department of Imaging Neuroscience).

***Electrical Pain Task.***

This task consisted of short (500 ms) painful and non-painful electrical stimuli delivered to the dorsum of the left hand above the first dorsal interosseous muscle. Every single trial consisted of an anticipation cue, indicating whether the upcoming stimulus would be painful, non-painful, or of uncertain quality. After a randomly jittered anticipation interval between 5 and 15 sec, stimulation was delivered by a Digitimer DS5 Isolated Bipolar Constant Current Stimulator (Digitimer Ltd., London, UK) via a surface electrode with 7mm diameter and a platinum pin (WASP electrode, Specialty Developments, Kent, UK). Since the quality of the stimuli in the empathy task was predictable (only painful expressions), we decided to ignore all uncertain conditions and focus the analysis of this task on the predictable painful and non-painful conditions. Four runs were performed, each containing five trials per condition. Before each session, participants’ pain thresholds and tolerance were individually determined to assess “non-painful but detectable” and “painful but tolerable” levels for use in the actual experiment (see 5, 6 for details on the calibration procedure). In this task, no subjective ratings were obtained. Due to technical problems, only 34 (of 35) healthy controls and 26 (of 29) acutely depressed patients completed both sessions of the electrical pain task.

**M4 – fMRI preprocessing**

fMRI was recorded with a 7 Tesla scanner (Siemens Magnetom, Siemens Medical Solutions, Erlangen, Germany) at the Medical University of Vienna, using a 32-channel head coil. 280 functional images were acquired during 390 seconds, using a single-shot gradient-recalled EPI-sequence (TR=1.4s, TE=23ms, matrix size 128 x 128 voxel, FOV of 192 x 192 mm, 78 slices of 1 mm with 0.25 mm gap). Multiband (factor 3) imaging with interleaved recording as well as optimized excitation pulses and readout bandwidths was used. Image preprocessing was carried out using a customized pipeline including tools from SPM, AFNI and FSL. The pipeline comprised (in this order) despiking, slice timing correction, bias field correction, realignment, normalization with Diffeomorphic Anatomical Registration Through Exponentiated Lie Algebra (DARTEL; (7)) and smoothing (6 mm Gaussian kernel). A 2 mm threshold for excessive head movements was applied. First-level and second-level analyses were performed with SPM12 (Wellcome Trust Centre for Neuroimaging, http://www.fil.ion.ucl.ac.uk/spm), adopting a general linear model approach.

**M5 – Statistical Analyses**

Statistical analyses of behavioral data were performed using SPSS 24.0 (Statistical Packages for the Social Sciences, v24.0). The level of significance was set to *p* < 0.05. All analyses met requirements of parametrical statistical tests (i.e., normality distribution and homogeneity of variances, as assessed by data inspection and statistical tests, i.e. Kolmogorow-Smirnow tests). Effect size estimates were calculated and interpreted as low, medium and large as suggested by Cohen (8).

***fMRI statistical analyses***

For all reported whole brain and connectivity analyses, the SPM extension “CorrClusTh.m” was used to calculate the corrected cluster size threshold (script by Thomas Nichols, University of Warwick, UK, and Marko Wilke, University of Tübingen, Germany; http://www2.warwick.ac.uk/fac/sci/statistics/staff/academic-research/nichols/scripts/spm/). A cluster-defining threshold of *p* < .001 together with a cluster probability of *p* < .05 FWE corrected for multiple comparisons was used.

**M6 – Whole brain analysis approach empathy task**

In order to check for possible additional effects in other brain regions, whole-brain cluster-level corrected analyses were run for the contrasts MDD: session 1 > session 2 (+ reverse contrast), Session 2: HC > MDD and Session 1: HC > MDD (+ reverse contrasts; initial height threshold = .001 uncorrected), as well as interaction contrasts (HC > MDD: S1 >S2; HC > MDD: S2 > S1; MDD > HC: S1 >S2; MDD > HC: S2 > S1). Because of absence of any interactions with the *condition* factor in the behavioral data and ROI analysis, and to increase power, effective and ineffective conditions were pooled for this analysis.

**Section R – Supplemental Results**

**R 1 – Empathy Task**

***Task Validation***

Before conducting the main analysis, we wanted to make sure that the empathy task we used led to the expected patterns of neural activation, which was done by a whole brain analysis of session 1 of the healthy controls (Contrast: Watching pain (effective & ineffective) > Baseline); *p*=.00001 uncorrected, as in the original paper by Lamm and colleagues; *k*=437). The resulting clusters covered similar regions as observed in Lamm, Batson (9): occipital cortex, insula, anterior midcingulate cortex, thalamus, basal ganglia (pallidum and caudate nucleus), bilateral periamygdalar region, cingulate and Supplemental motor area, lateral precentral gyrus, temporo-parietal and lateral prefrontal areas. See Supplemental table T1 for details.

***Whole-brain analysis***

Whole-brain analysis of task-related treatment effects showed no significant clusters in the contrast MDD S2 > S1. The reverse contrast (MDD S1 > S2) revealed clusters in the left anterior insula, Supplemental motor area, anterior midcingulate cortex and occipital cortex. Comparing HC > MDD in the baseline session yielded two clusters in the bilateral lateral occipital cortex and one in the right posterior superior temporal sulcus. After treatment, more clusters were detected in the group comparison (S2: HC > MDD): temporoparietal junction, ventrolateral prefrontal cortex, orbitofrontal cortex, fusiform face area, brainstem, medial prefrontal cortex, Supplemental motor area. The reverse contrasts (MDD > HC) resulted in one cluster in the post-treatment session (secondary somatosensory cortex) and four clusters in the baseline session (left occipital cortex, secondary somatosensory cortex, left posterior superior temporal sulcus). Interaction contrasts across sessions and groups (e.g., HC > MDD: S2 > S1) did not yield any significant clusters. See Supplemental tables T2 & T3 for details.

**R2 – Electrical Pain Task**

Follow-up analysis of *ROI * intensity* interaction in the four-way mixed model ANOVA:

To test whether this effect was driven by smaller differences between pain and no-pain in the aMCC, we computed mean values of both pain and no-pain values across sessions for each individual ROI, subtracted them from each other (pain - no-pain per ROI) and computed *t*-tests between ROIs on these values: aMCC vs. lAI: *t*(59)=4.88, *p*<.001; aMCC vs. rAI: *t*(59)=5.73, *p*<.001; lAI vs. rAI: *t*(59)=-0.07, *p*=.946.

***Single ROI ANOVAs***

**Left anterior Insula:** The three-way mixed model ANOVA revealed a significant main effect of *intensity* (*F*(1,58)=26.87, *p*<.001, *η^2^_p_* = .317; higher values in response to pain compared to no-pain) and a significant interaction of *session* * *intensity * group* (*F*(1,58)=4.11, *p*=.047, *η^2^_p_*=.066), driven by the HC group showing an increase in values in response to no-pain stimuli. All other main effects and interactions remained non-significant (all *p*-values > .142).

**Right anterior Insula:** The three-way mixed model ANOVA revealed a significant main effect of *intensity* (*F*(1,58)=33.33, *p*<.001, *η^2^_p_* = .365; higher values in response to pain compared to no-pain) and a trend for an interaction of *session* * *intensity * group* (*F*(1,58)=3.59, *p*=.063, *η^2^_p_*=.058). All other main effects and interactions remained non-significant (all *p*-values > .237).

**Anterior midcingulate cortex:** The three-way mixed model ANOVA revealed a significant main effect of *intensity* (*F*(1,58)=7.76, *p*=.007, *η^2^_p_* = .118; higher values in response to pain compared to no-pain) and a trend for an interaction of *session* * *intensity * group* (*F*(1,58)=3.69, *p*=.060, *η^2^_p_*=.060). All other main effects and interactions remained non-significant (all *p*-values > .215).

***Whole brain analysis***

For a whole-brain analysis of the pain task, see Kraus, Klöbl (10). In short, this analysis revealed reduced pre-treatment activity in the MDD group compared to the HC group in the pulvinar nuclei of the thalamus. This effect was restricted to the no-pain condition and vanished after treatment.

***Pain calibration thresholds***

Regarding pain calibration threshold changes across sessions, the repeated measures ANOVA only showed an expected main effect of intensity (*F*(1,58)=72.36, *p*<.001, *η^2^_p_* = .555), reflecting lower no-pain compared to pain values (see Supplemental table T4 for means). All other main effects and interactions were non-significant (all *p*-values > .40).

**R3 – Questionnaires on empathy and emotion**

In ERQ Suppression, a significant main effect of *group* (*F*(1,53)=9.31, *p*=.004, *η^2^_p_* = .149) was found, which was driven by a higher tendency to suppress negative emotions in patients in both sessions. In ERQ Reappraisal, significant main effects of *session* (*F*(1,53)=4.76, *p*=.034, *η^2^_p_* = .082) and *group* (*F*(1,53)=4.52, *p*=.038, *η^2^_p_* = .079) were found, as well as an interaction of *session* * *group* (*F*(1,53)=4.76, *p*=.034, *η^2^_p_* = .082). This interaction was driven by a significantly lower tendency to use reappraisal as a strategy for emotion regulation in the MDD compared to the HC group in the first, but not in the second session. For ECS Fear, a significant main effect of *session* (*F*(1,58)=6.36, *p*=.014, *η^2^_p_* = .099) was found, as well as a significant main effect of *group* (*F*(1,58)=7.81, *p*=.007, *η^2^_p_* = .119). This was driven by higher values in the MDD group in general and a decrease in both groups from the first to the second session. Significant main effects of *group* were found for ECS Joy (*F*(1,58)=24.35, *p*<.001, *η^2^_p_* = .296) and ECS Love (*F*(1,58)=7.53, *p*=.008, *η^2^_p_* = .115), both driven by higher values in the control group, as well as for ECS Anger (*F*(1,58)=4.46, *p*=.039, *η^2^_p_* = .072), driven by higher values in the MDD group. For ECS sadness, we found a significant *session * group* interaction (*F*(1,58)=15.18, *p*<.001, *η^2^_p_* = .208) driven by a decrease from session 1 to session 2 only in the MDD group. For IRI Personal Distress, a significant main effect of *group* (*F*(1,58)=44.62, *p*<.001, *η^2^_p_* = .435) was found, driven by substantially higher distress values in the MDD group in both sessions. IRI Empathic Concern, Fantasy and Perspective Taking scales did not show any significant main effects or interactions.

**R4 – Effects of remission on post-treatment behavioral/neural empathic responses**

No significant correlations between post-treatment HAM-D scores and empathic responses were found (all p-values > .114).

**R5 – Effects of MDD history on behavioral/neural empathic responses**

No significant correlations between duration of disease or number of episodes with empathic responses were found (all p-values > .112).

**R6 – Effects of improvements in ERQ Reappraisal on behavioral/neural empathic responses**

All repeated measures ANOVA main effects and interactions were largely unaffected by including improvements in ERQ Reappraisal (S1-S2).

**Section T - Supplemental Tables**

*Supplemental table T1.* Task validation. Whole-brain activation in the HC group, session 1 (Contrast: Watching pain (effective & ineffective) > Baseline); *p*=.00001 uncorrected, cluster size threshold = 427). Results are in line with Lamm et al. 2007.

| **Brain region(s)** | **k** | **peak x** | **peak y** | **peak z** | ***t* value** | ***p* value (FWE-corr.)** |
| --- | --- | --- | --- | --- | --- | --- |
| *Whole-brain analysis* | | | | | | |
| R visual cortex | 32971 | 48 | -73 | 1 | 36.97 |  |
| L visual cortex | 13511 | -45 | -79 | -2 | 34.36 |  |
| L Amygdala / periamygdalar regions / insula | 8978 | -20 | -10 | -14 | 16.09 |  |
| Supplemental motor area / anterior midcingulate cortex | 3421 | 2 | 14 | 61 | 11.74 |  |
| Medial prefrontal cortex | 2357 | 6 | 53 | 31 | 9.62 |  |

*Supplemental table T2*. Whole-brain activation clusters in the contrasts MDD: S1 > S2 and MDD: S2 > S1 (*p*=.001 uncorrected, cluster size threshold = 427).

| **Brain region(s)** | **k** | **peak x** | **peak y** | **peak z** | ***t* value** | ***p* value (FWE-corr.)** |
| --- | --- | --- | --- | --- | --- | --- |
| ***MDD: S1 > S2*** | |  |  |  |  |  |
| Supplemental motor area / anterior midcingulate cortex | 2693 | 14 | 27 | 60 | 4.51 | <.001 |
|  |  | 0 | 44 | 39 | 4.12 |  |
|  |  | 3 | 35 | 46 | 3.97 |  |
| Left anterior insula | 1524 | -50 | 18 | 7 | 4.43 | <.001 |
|  |  | -57 | 17 | 16 | 4.31 |  |
|  |  | -36 | 21 | -6 | 4.25 |  |
| Occipital cortex | 460 | 22 | -90 | 30 | 4.28 | .038 |
|  |  | 20 | -88 | 39 | 4.10 |  |
| ***MDD: S2 > S1*** | |  |  |  |  |  |
| - | - | - | - | - | - | - |

*Supplemental table T3*. Whole-brain activation clusters in the contrasts S1: HC > MDD and S2: HC > MDD (and reverse contrasts; *p*=.001 uncorrected, cluster size threshold = 427) and interaction contrasts (HC > MDD: S1>S2 etc.). Only the highest peak is reported in case of several confluent peaks.

| **Brain region(s)** | **k** | **peak x** | **peak y** | **peak z** | ***t* value** | ***p* value (FWE-corr.)** |
| --- | --- | --- | --- | --- | --- | --- |
| ***S1: HC > MDD*** | |  |  |  |  |  |
| Right lateral occipital cortex | 1970 | 50 | -73 | 4 | 8.71 | <.001 |
| Left lateral occipital cortex | 2863 | -42 | -78 | 3 | 4.73 | <.001 |
| Right posterior superior temporal sulcus | 1514 | 50 | -37 | 15 | 7.18 | <.001 |
| ***S2: HC > MDD*** | |  |  |  |  |  |
| Temporoparietal junction | 3924 | 64 | -45 | 16 | 8.82 | <.001 |
| Occipital cortex | 18069 | -15 | -93 | 27 | 8.03 | <.001 |
| Inferior frontal gyrus (?) | 2178 | 59 | 18 | 28 | 6.47 | <.001 |
| Temporal lobe | 685 | 57 | 8 | -20 | 6.42 | .007 |
| Ventrolateral prefrontal cortex | 4081 | -51 | 30 | 19 | 6.06 | <.001 |
| Anterior prefrontal cortex | 1024 | -26 | 51 | 19 | 5.55 | .001 |
| Orbitofrontal cortex | 824 | -39 | 33 | -20 | 5.51 | .003 |
| Fusiform face area | 508 | -33 | -45 | -17 | 5.12 | .026 |
| Brainstem | 579 | -6 | -31 | -5 | 5.06 | .015 |
| Medial prefrontal cortex | 1609 | 6 | 42 | -6 | 4.88 | <.001 |
| Supplemental motor area | 1900 | 8 | 20 | 66 | 4.62 | <.001 |
| ***S1: MDD > HC*** | |  |  |  |  |  |
| Left occipital cortex | 514 | -32 | -84 | 42 | 5.53 | .025 |
| Secondary somatosensory cortex | 431 | 60 | 6 | 1 | 5.05 | .048 |
| Secondary somatosensory cortex | 1094 | 45 | -30 | 21 | 4.76 | .001 |
| Left posterior superior temporal sulcus | 574 | -45 | -51 | 10 | 4.90 | .016 |
|  | 466 | 45 | -58 | 34 | 4.76 | .037 |
| ***S2: MDD > HC*** | |  |  |  |  |  |
| Secondary somatosensory cortex | 540 | 54 | -24 | 21 | 5.12 | .021 |
|  |  |  |  |  |  |  |
| ***HC > MDD (S2 > S1)*** | |  |  |  |  |  |
| - |  |  |  |  |  |  |
| ***HC > MDD (S1 > S2)*** | |  |  |  |  |  |
| - |  |  |  |  |  |  |
| ***MDD > HC (S2 > S1)*** | |  |  |  |  |  |
| - |  |  |  |  |  |  |
| ***MDD > HC (S1 > S2)*** | |  |  |  |  |  |
| - |  |  |  |  |  |  |

*Supplemental table T4.* Pain Calibration values. Values are mean mA ± S.E.M.

| **Group** | **Session 1** | | **Session 2** | |
| --- | --- | --- | --- | --- |
|  | *No-pain* | *Pain* | *No-pain* | *Pain* |
| **HC** | .12±.02 | .55±.09 | .16±.04 | .55±.10 |
| **MDD** | .15±.02 | .49±.06 | .14±.02 | .50±.06 |

**Supplemental References**

1. First MB, Benjamin LS, Gibbon M, Spitzer RL, Williams JB (1997): *Structured clinical interview for DSM-IV Axis II personality disorders*. American Psychiatric Press.

2. First MB, Spitzer RL, Gibbon M, Williams JB (2002): Structured clinical interview for DSM-IV-TR axis I disorders, research version, patient edition. SCID-I/P.

3. Zimmerman M, Martinez JH, Young D, Chelminski I, Dalrymple K (2013): Severity classification on the Hamilton depression rating scale. *Journal of affective disorders*. 150:384-388.

4. Faul F, Erdfelder E (1992): GPOWER: A priori, post-hoc, and compromise power analyses for MS-DOS [Computer program]. *Bonn, FRG: Bonn University, Department of Psychology*.

5. Hahn A, Kranz GS, Seidel E-M, Sladky R, Kraus C, Küblböck M, et al. (2013): Comparing neural response to painful electrical stimulation with functional MRI at 3 and 7 T. *NeuroImage*. 82:336-343.

6. Seidel EM, Pfabigan DM, Hahn A, Sladky R, Grahl A, Paul K, et al. (2015): Uncertainty during pain anticipation: the adaptive value of preparatory processes. *Human brain mapping*. 36:744-755.

7. Ashburner J (2007): A fast diffeomorphic image registration algorithm. *Neuroimage*. 38:95-113.

8. Cohen J (1988): Statistical power analysis for the behavioral sciences 2nd edn. Erlbaum Associates, Hillsdale.

9. Lamm C, Batson CD, Decety J (2007): The neural substrate of human empathy: effects of perspective-taking and cognitive appraisal. *Journal of cognitive neuroscience*. 19:42-58.

10. Kraus C, Klöbl M, Tik M, Auer B, Vanicek T, Geissberger N, et al. (2018): The pulvinar nucleus and antidepressant treatment: dynamic modeling of antidepressant response and remission with ultra-high field functional MRI. *Molecular psychiatry*.1.
